# Characteristics of clinical studies of summer acupoint herbal patching: a bibliometric analysis

**DOI:** 10.1186/s12906-015-0905-z

**Published:** 2015-10-22

**Authors:** Fen Zhou, Dan Yang, Jing-yu Lu, Yan-fu Li, Kai-yue Gao, Ya-jing Zhou, Ruo-xue Yang, Juan Cheng, Xiao-xiong Qi, Lily Lai, George Lewith, Jian-ping Liu

**Affiliations:** Center for Evidence-Based Chinese Medicine, Beijing University of Chinese Medicine, 11 Bei San Huan Dong Lu, Chaoyang District, Beijing 10029 China; School of Nursing, Beijing University of Chinese Medicine, Beijing, 10029 China; Complementary and Integrated Medicine Research Unit, Primary Care and Population Sciences, University of Southampton, Southampton, UK

**Keywords:** Summer acupoint herbal patching, Sanfutie, Traditional Chinese therapies, Bibliometrics, literature review

## Abstract

**Background:**

Summer acupoint herbal patching (SAHP) has been widely used in China for thousands of years. This bibliometric analysis aims to provide a comprehensive review of the characteristics of clinical studies on SAHP for any condition.

**Methods:**

We included clinical studies such as randomized clinical trials (RCTs), controlled clinical studies (CCTs), case series (CSs), case reports (CRs), and cross-sectional studies on SAHP for any condition. Six databases were searched from date of inception to March 2015. Bibliometric information and study details such as study type, characteristics of participants, details of the intervention and comparison, and outcome were extracted and analyzed.

**Results:**

A total of 937 clinical studies were identified and which were published between 1977 and 2015. This included 404 RCTs, 52 CCTs, 458 CSs, 19 CRs and 4 cross-sectional studies and involved 232,138 participants aged 2 to 90 years from two countries. Almost all studies were from China (936, 99.89 %). The five conditions most commonly treated by SAHP were asthma (401, 42.80 %), chronic bronchitis (146, 15.58 %), allergic rhinitis (117, 12.49 %), chronic obstructive pulmonary disease (73, 7.79 %), and recurrent respiratory tract infection (42, 4.48 %). Among 502 controlled studies, the majority compared SAHP alone with different controls (16 categories, 275 comparisons). The most commonly used controls were western medicine, placebo, traditional Chinese medicine, no treatment and non-pharmaceutical traditional Chinese therapies. Composite outcome measures were the most frequently reported outcome (512, 69.19 %).

**Conclusion:**

A substantial amount of research on SAHP has been published in China and which predominantly focuses on respiratory conditions. The findings from this study can be used to inform further research by highlighting areas of greatest impact for SAHP.

**Electronic supplementary material:**

The online version of this article (doi:10.1186/s12906-015-0905-z) contains supplementary material, which is available to authorized users.

## Background

Acupoint herbal patching (AHP) is the application of herbal preparations to acupuncture points and is used for the prevention, as well as treatment, of various conditions in traditional Chinese medicine (TCM). The use of AHP was first recorded in the Prescriptions for Fifty-two Diseases (*Wu Shi Er Bing Fang*), written in approximately the fourth century BC and considered the oldest herbal text in TCM [1]. A variation of AHP exists called summer acupoint herbal patching (SAHP) which refers to AHP applied only during the summer and which was first described in the *Zhang Shi Yi Tong* text by *Zhang Lu* (*Qing* Dynasty, 1644–1912) [2]*.* SAHP is also known as *sanfutie* in Chinese and is commonly applied during *sanfu* in the lunar calendar [3]. *Sanfu* refers to three specific periods in the lunar calendar and it is during these three periods (each includes ten days) that SAHP is applied [4]. Since the 1950s, *Zhang Lu*’s classic approaches have been applied in modern clinical contexts and SAHP has become increasingly popular in China owing to its convenience and non-invasive nature [2]. In 2014, the State Administration of Traditional Chinese Medicine reported the use of SAHP in 620 medical institutions in Beijing, an increase of 13.14 % compared with 2013 [5, 6].

SAHP is considered a complex therapy as it uses both herbal medicine and acupuncture points. During SAHP, herbs with warm and acrid properties are thought in TCM to be more likely absorbed percutaneously and which in turn stimulates the meridian *qi*. Animal experiments suggest that the mechanism of AHP may be related to reducing serum IgE and IL-4 levels, restraining the release of inflammatory mediators, and adjusting the expression level of transcription factors in the rat model of asthma [7, 8]. This purpose of AHP is to regulate the functions of the body and strengthen the self-healing capacity of the body so as to prevent and treat disease [9–11]. However in SAHP, this effect is further enhanced through the specific application during *sanfu* based on the TCM treatment principle of ‘*treating winter disease in summer’*. The rationale behind this treatment principle is that some conditions occur during winter predominantly due to a deficiency of *yang qi*, levels of which peak during the summer and especially during the *sanfu*. Thus TCM theory holds that by applying AHP during these 3 days, patients can maximize *yang qi* and in turn prevent recurrence of a winter condition [3].

Owing to its growing popularity, we are interested in exploring SAHP further as a potential treatment. To assist in developing appropriate areas of investigation and to formulate relevant research questions, we wished to initially identify which conditions in particular SAHP appears to be used for, and furthermore to obtain an overview of the study designs used. To date, 3 Chinese-language articles have been published in 2010 but which only analyze selected acupoints and herbal ingredients of SAHP [12–14]. Since there was a limited scope within these publications, only a minimal amount of information is presented regarding application of SAHP. Our aim in this study therefore was to conduct a comprehensive bibliometric analysis of all clinical studies featuring SAHP.

## Methods

### Eligibility criteria

We included all types of clinical studies including randomized controlled trials (RCTs), clinical controlled trials (CCTs), cross-sectional, case series (CSs), and case reports (CRs). Reviews, editorials, popular science papers, and letters to the editor were not eligible. No limitations were placed in terms of language or publication type. Where multiple publications reporting the same data were found, we included only the first publication in our analysis.

Studies were eligible if they evaluated the therapeutic effect or safety of SAHP. AHP studies that were conducted outside of the summer period were excluded. We placed no limitations on the types of disease or condition, nor on the herbal ingredients, acupoints, or time of SAHP application.

### Comparisons

For RCT and CCT studies, acceptable comparisons were no treatment, placebo, western medication, and other TCM therapy. Combined therapy with SAHP and other interventions compared with other interventions alone were also included. Comparisons consisting of variations of SAHP were also included, such as SAHP versus AHP, or different application times, preparation types or herbal formulae.

### Identification and selection of studies

We searched six Chinese and English language electronic databases from inception to March 11 2015: China Network Knowledge Infrastructure (CNKI) (1911–1978, 1979–2015) Chinese Scientific Journal Database (VIP) (1989–2015), Wan Fang Database (1985–2015), Chinese Biomedical Literature Database (Sino-Med) (1978–2015), PubMed (1966–2015) and the Cochrane Library (Issue 3, 2015). Some of these databases also include unpublished sub-databases: CNKI includes China Doctoral Thesis Full-text Database, China Masters’ Thesis Full-text Database, International Conference Proceedings Database, and National Key Conference Proceedings Database; while Wanfang database contains Wanfang Patent Database, Database of Standards. Our search terms included “SAHP, *sanfutie*, *tianjiu*”. The search strategies for each database are shown in Additional file 1.

Six review authors were involved in study identification and selection. FZ first screened titles and abstracts using NoteExpress 2.9.8 and five authors (YJZ, YFL, DY, KYG and JYL) then selected eligible studies. Each of the five authors were assigned one fifth of the full text articles to conduct study eligibility independently. FZ checked the eligibility of each included studies again.

### Data extraction and analysis

We developed an electronic data extraction form using Epidata 3.1, the extracted data are shown in Additional file 2. This was piloted by eight authors (YFL, DY, KYG, JYL, YJZ, RXY, JC, XXQ) who extracted data from a sample of 15 articles during the piloting phase. Data extraction was then conducted by the same eight authors for the remaining publications by dividing the retrieved publications equally. One author (FZ) checked the extracted data.

The extracted variables included full citations, study design, participants (origin, sample size, gender, age, and medical condition), details of SAHP (herbal ingredients, acupoints, treatment session, treatment course, frequency), control treatments and outcome (follow up, outcome type). Disagreements regarding study eligibility and data extraction were discussed with one author (JPL).

These data were imported into SPSS and analyzed descriptively using SPSS® (release 20.0, IBM, Armonk, NY, USA) and Microsoft Excel (version 12.3.5, Microsoft, Redmond, WA, USA). Descriptive statistics was based on the type of variables. Categorical variables were reported as frequencies and percentages. For continuous variables, we used means and standard deviations for parametric data and reported median and range for non-parametric data.

### Ethics

Since this study drew from publicly available data and did not recruit human participants, ethical approval was not required.

## Results

### General description of overall studies

**Fig. 1:**
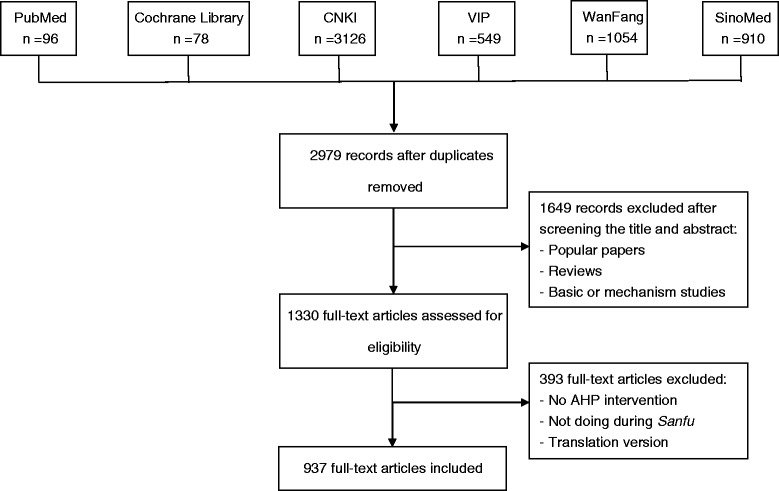
The Flowchart of including and excluding studies

A total of 5987 records were identified and abstracts screened. Of these, the majority were excluded due to duplication or being clearly irrelevant from the title or abstract. Full texts for the remaining 1330 records were retrieved to assess eligibility further and which eventually enabled 937 articles to be included for our analysis. Of these, 932 (99.47 %) articles were published in Chinese whilst 5 (0.53 %) were published in English. 31 (3.31 %) publications were conference full text papers and 29 (3.10 %) were dissertations (Fig. 1).

**Fig. 2:**
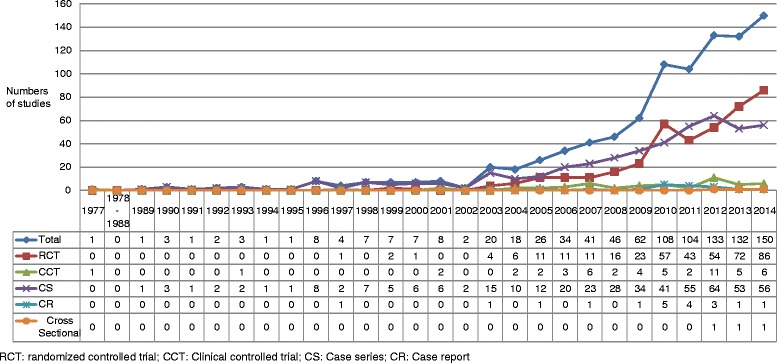
Numbers of studies on *S*AHP by study type between 1977 to 2014

The earliest publication was in 1977, which compared SAHP with no treatment for chronic bronchitis and which involved 40 patients from the community [15]. The second earliest publication was identified as a CS, published in 1989 and which involved 580 asthma and chronic bronchitis patients [16]. In terms of the quantity of publications, this appeared to increase after 2003 (Fig. 2). Between 2009 and 2010, the number of articles published almost doubled. Following 2010, increase tendency in publications became relatively stable between 2011 and 2014 with a peak of 151 reports in 2014. For the year 2015, 7 studies involving 6 RCTs and one cross-sectional study were identified. However, since the year of 2015 was not yet completed at the time of our literature search, we did not display data for 2015 in Fig. 2.

**Fig. 3:**
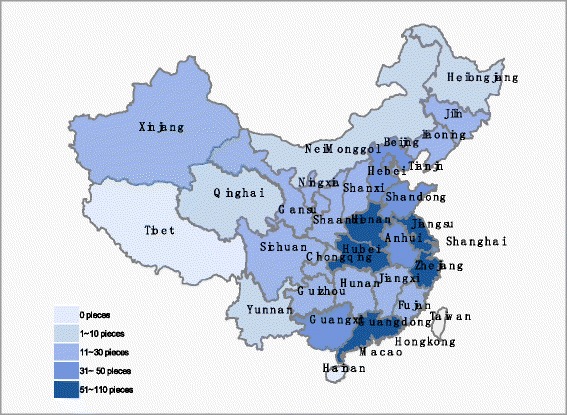
Data map of SAHP studies in China

Of the 937 studies, one was conducted in Switzerland, while the remaining studies were performed in China. The 936 analyzed articles originated in almost over China except to Hainan province, with top five provinces coming from Guangdong (108, 11.53 %), Hubei (90, 9.61 %), Zhejiang (74, 7.90 %), Jiangsu (73, 7.79 %), Henan (66, 7.04 %) (Fig. 3).

Out of the 937 studies, 60 (6.40 %) were conference papers and dissertations. The remaining 877 (93.60 %) reports were published in 213 different journals; with the highest frequency of publications in Chinese-language journals: *Shanghai Journal of Acupuncture and Moxibustion* (31, 3.53 %), *Guangming Journal of Chinese Medicine* (29, 3.31 %), *Hubei Journal of Traditional Chinese Medicine* (22, 2.51 %), *Chinese Acupuncture & Moxibustion* (19, 2.17 %), *Journal of Clinical Acupuncture and Moxibustion* (18, 2.05 %), *Liaoning Journal of Traditional Chinese Medicine* (17, 1.94 %), *Journal of New Chinese Medicine* (17, 1.94 %), *Shanxi Journal of Chinese Medicine* (17, 1.94 %), and *Zhejiang Journal of Traditional Chinese Medicine* (16, 1.82 %). Only 5 studies had been published in international journals: *American Journal of Chinese Medicine* (1 study) [17], *Evidence-based Complementary and Alternative Medicine* (2 studies) [18, 19], and *Journal of Traditional Chinese Medicine* (2 studies) [20, 21].

### Study design

Amongst the 937 studies, there were 404 RCTs (43.12 %), 52 CCTs (5.55 %), 458 CSs (48.88 %), 19 CRs (2.03 %) and 4 cross-sectional studies (0.43 %). The first RCT was published in 1997. 36 (3.84 %) studies were reported as being multi-centered. When combining RCTs and CCTs together, the majority (412, 90.35 %) featured 2 treatment arms, 39 (8.55 %) were designed as 3 arms, and 5 (1.10 %) featured four arms.

### Disease categories

The 937 studies included a total of 232,138 participants and who were recruited from a number of settings including the following: hospital (884, 94.34 %), community health center (16, 1.71 %), clinic (2, 0.21 %) and nursing home (1 study). 34 (3.63 %) studies did not provide details of setting. Study sample sizes of RCTs and CCTs ranged from 19 to 1042. Thirty studies (3.20 %) did not report the participants’ age. Most studies (696, 74.28 %) included adults (>18 years), 183 (19.53 %) studies only enrolled children and /or adolescents (<18 years), 7 studies enrolled older adults (>60 years), and 28 (2.99 %) studies involved participants of different ages. Among the 772 studies which reported gender, the proportion of male (52.57 %) and female (47.43 %) participants were almost equal.

Amongst the studies, thirty-five different conditions were treated and which can be seen in Table 1. The most frequently reported were respiratory disorders with 742 (79.19 %) studies. The 10 highest ranking conditions where SAHP was most frequently applied were asthma (401, 42.80 %), chronic bronchitis (146, 15.58 %), allergic rhinitis (117, 12.49 %), chronic obstructive pulmonary disease (73, 7.79 %), recurrent respiratory tract infection (42, 4.48 %), cough and panting (42, 4.48 %), knee osteoarthritis (23, 2.45 %), rheumatoid arthritis (15, 1.60 %), common cold (8, 0.85 %), and sub-health (a condition characterized by some disturbances in psychological or physical characteristics but with no typical pathologic features) (7, 0.75 %). The remaining 25 conditions were found in less than 6 studies and among these 6, one study alone featured 13 conditions. 9 studies did not report a condition since the focus of the study was on side effects or adverse events (Table 1).

**Table 1:** Number of condition categories in clinical SAHP studies from 1977 to 2015 (*n* = 928)

Disease/condition	Frequency	Percentage	Disease/condition	Frequency	Percentage
Asthma	401	43.21	Chronic Pelvic Inflammation	2	0.22
Chronic Bronchitis	146	15.73	Chronic Renal Failure	2	0.22
Allergic Rhinitis	117	12.61	Chronic Pulmonary Heart Disease	2	0.22
Chronic Obstructive Pulmonary Disease	73	7.87	Dysmenorrhea	2	0.22
Recurrent Respiratory Tract Infection	42	4.53	Postpartum Bodily Pain	1	0.11
Cough and panting	42	4.53	Pneumoconiosis	1	0.11
Knee Osteoarthritis	23	2.48	Tourette’s syndrome	1	0.11
Rheumatoid Arthritis	15	1.62	Night Sweat	1	0.11
Common Cold	8	0.86	Chronic Glomerulonephritis	1	0.11
Sub-health	7	0.75	Coronary Heart Disease	1	0.11
Irritable Bowel Syndrome	6	0.65	Rachitis	1	0.11
Chronic Pharyngitis	5	0.54	Scapulohumeral Periarthritis	1	0.11
Ankylosing Spondylitis	4	0.43	Insomnia	1	0.11
Diarrhea	4	0.43	Diabetic Nephropathy	1	0.11
Dyspepsia	4	0.43	Psoriasis	1	0.11
Chilblains	4	0.43	Primary Glomerulonephritis	1	0.11
Cervical Spondylosis	3	0.32	Lumbar Osteoarthritis	1	0.11
Pneumonia	3	0.32			

### Intervention categories

A total of 890 studies (94.98 %) reported sufficient information regarding the season in which SAHP was applied and treatment duration. In terms of the SAHP application, 787 (88.43 %) studies reported applying only during *sanfu* period, 103 (11.70 %) studies reported applying during both *sanfu* and *sanjiu* period (winter season). The remainder of the 47 studies mentioned applying SAHP only during the summer. The most common treatment course was one *sanfu* and which was reported in 443 studies (50.34 %). 10 *sanfu*s was the longest treatment course reported in 1 study [22], with a median length of 1 *sanfu*. The number of SAHP sessions per *sanfu* were reported in a total of 871 studies (range: 3 to 40, median = 3 sessions), with the majority reporting 3 sessions (599 studies, 68.77 %).

Asthma was the most commonly treated condition from the included studies. We focused our analysis of SAHP acupoints and herbal ingredients on the 401 asthma studies. Complete information of acupoints and herbal ingredients were available in 386 (95.55 %, reporting 1736 acupoints) and 362 (89.60 %, reporting 1833 herb ingredients) studies respectively. A total of 42 acuponts were selected for SAHP and the five most commonly used acupoints were: BL13, EX-B1, GV14, RN22 and BL17. One hundred and two herbal ingredients were reported and the five most frequently used herbs were: *Semen Sinapis (baijizi)*, *Herba Asari (xixin)*, *Radix Kansui (gansui)*, *Rhizoma Corydalis (yanhusuo)* and *Herba Ephedrae (mahuang)* (Table 2).

**Table 2:** Details SAHP intervention. Frequency and percentage of details applied season, treatment course, treatment sessions per *sanfu*, herb ingredient, acupoint selected of SAHP intervention

Details of SAHP intervention	Frequency	Percentage	Details of SAHP intervention	Frequency	Percentage	Details of SAHP application	Frequency	Percentage
Applied season (890 studies with sufficient information)			Herb ingredient (362 studies with sufficient information)			Herb ingredient (362 studies with sufficient information)		
Only in *sanfu*	787	88.43	* Semen Sinapis*	351	96.96	* Radix et Rhizoma Salviae Miltiorrhizae*	5	1.38
Combined with *sanjiu*	103	11.57	* Radix et Rhizoma Asari*	324	89.50	* Lignum Santali Albi*	5	1.38
Treatment course (890 studies with sufficient information)			*Radix Kansui*	297	82.04	Others	84	23.20
1 *sanfu*	453	50.90	* Rhizoma Corydalis*	269	74.31			
2 *sanfus*	40	4.49	* Herba Ephedrae*	80	22.10	Acupoint selected (386 studies with sufficient information)		
3 *sanfus*	289	32.47	* Cortex Cinnamomi*	57	15.75	BL 13	374	96.89
5 *sanfus*	4	0.45	* Moschus*	54	14.92	EX-B1	195	50.52
10 *sanfus*	1	0.11	* Rhizoma Pinelliae*	44	12.15	DU 14	160	41.45
1 *sanfu and sanjiu*	61	6.85	* Borneolum Syntheticum*	27	7.46	RN 22	140	36.27
2 *sanfus and sanjius*	9	1.01	* Flos Caryophylli*	25	6.91	BL 17	130	33.68
3 *sanfus and sanjius*	33	3.71	* Radix Aconiti Lateralis Praeparata*	24	6.63	BL 15	118	30.57
Treatment sessions per *sanfu* (871 studies with sufficient information)			*Fructus Perillae*	18	4.97	RN 17	117	30.31
4 times	45	5.17	* Radix Angelicae Dahuricae*	14	3.87	BL 23	114	29.53
5 times	43	4.94	* Pheretima*	13	3.59	BL 43	104	26.94
6 times	86	9.87	* Fructus Evodiae*	12	3.31	BL 20	88	22.80
7 times	2	0.23	* Radix Saposhnikoviae*	10	2.76	BL 12	64	16.58
8 times	15	1.72	* Radix et Rhizoma Asari*	10	2.76	ST 36	25	6.48
9 times	34	3.90	* Pericarpium Zanthoxyli*	9	2.49	EX-HN15	17	4.40
10 times	14	1.61	* Flos Daturae*	9	2.49	BL 11	15	3.89
12 times	6	0.69	* Fructus Gleditsiae*	9	2.49	ST 40	10	2.59
15 times	7	0.80	* Radix Astragali*	8	2.21	BL 21	7	1.81
16 times	2	0.23	* Mylabris*	7	1.93	LU 1	7	1.81
18 times	6	0.69	* Semen Raphani*	7	1.93	BL 52	6	1.55
20 times	3	0.34	* Fructus Schisandrae Chinensis*	7	1.93	BL 14	5	1.30
24 times	3	0.34	* Lignum Aquilariae Resinatum*	6	1.66	RN 6	5	1.30
28 times	1	0.11	* Rhizoma Chuanxiong*	6	1.66	Others	31	8.03
30 times	2	0.23	* Radix Scutellariae*	6	1.66			
40 times	3	0.34	* Radix Stemonae*	5	1.38			

### Comparison types

**Fig. 4:**
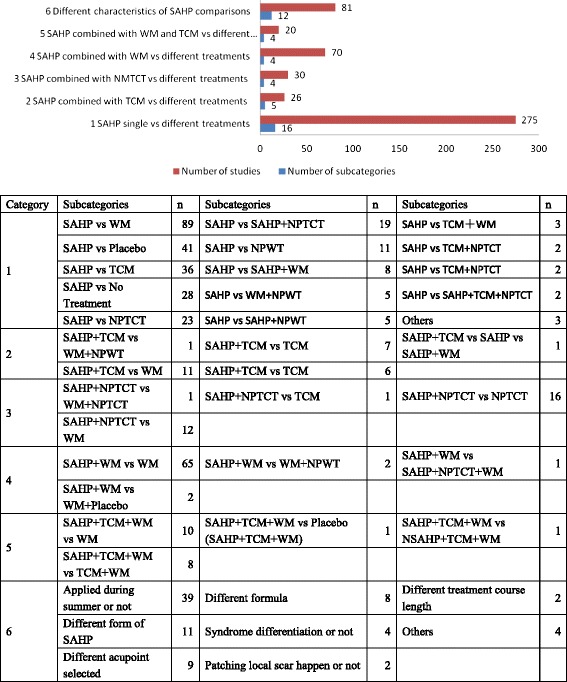
Number of different comparison categories and subcategories in clinical studies. *n* = number of comparisons; others = subcategory that only appear once. WM: Western medicine; TCM: traditional Chinese medicine; NPTCT: Non pharmaceutical traditional Chinese therapy; NPWT: Non pharmaceutical western therapy

A total of 502 comparisons were reported in the 485 controlled clinical studies and which are shown in Fig. 4. These 502 comparisons were classified into 6 different comparisons: SAHP alone *vs* different treatments (16 subcategories, 275 comparisons), SAHP combined with other TCM therapies *vs* different treatments (5 subcategories, 26 comparisons), SAHP combined with non pharmaceutical traditional Chinese therapy *vs* different treatments (4 subcategories, 30 comparisons), SAHP combined with western medicine *vs* different treatments (4 subcategories, 70 comparisons), SAHP combined with western medicine and TCM *vs* different treatments (4 subcategories, 20 comparisons), different characteristics of SAHP comparisons (12 subcategories, 81 comparisons, patching during *sanfu* or not, different form of SAHP, acupoint selected, formula, etc.). As no comparisons are reported in CS, CR and cross-sectional studies, we analyzed the RCTs and CCTs for details on comparison treatments. The details of SAHP comparison categories can be found in the Fig. 4.

### Outcomes

Of the 937 studies, 740 studies reported sufficient information regarding the outcome measure. 373 (50.41 %) studies stated that the outcome was evaluated immediately after SAHP; and 321 (43.38 %) studies evaluated the effects after a follow up period; the remaining 46 (6.22 %) studies reported measuring the outcome post SAHP and at follow up. The reported follow-up frequency ranged from 1 to 5 times (once a year, a total of five years), with a median of once; whereas the follow-up period ranged from 10 days to 36 months, with a median of 12 months.

**Fig. 5:**
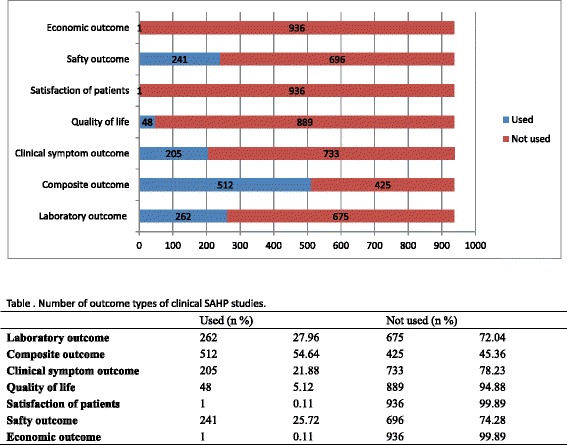
Number of outcome types of clinical SAHP studies

The most frequently reported outcome measures were laboratory indices (262 studies, 35.41 %), followed by safety (241 studies, 32.57 %), clinical symptom (205 studies, 27.70 %), quality of life (48 studies, 6.49 %), satisfaction of patients (1 study) and economic index (1 study). 512 studies (69.19 %) used composite outcome measures for clinical effectiveness by incorporating several outcome components into one comprehensive category (Fig. 5).

## Discussion

This bibliometric analysis has comprehensively identified and analyzed clinical studies of SAHP, and included 937 reports mainly from China with one study from Switzerland [23]. Whilst the earliest study did not commence until 1977, the majority appeared after 2003 with multi-fold increase in RCTs and CSs. The quantity of studies on SAHP has increased during the last 40 years. Although there was a slight reduction in 2011 of publications in SAHP, the overall trend appears to be that the number of studies is continuing to increase. The total number of studies published in 2014 was three times more than that in 2008. In addition, there were 5 studies published in 3 international journals [17–21]; suggesting that SAHP, a therapy conducted predominantly in China, is being promoted to an international audience.

CS was the most frequently used study design (48.88 %). This may be due to the ease of conducting a CS since the absence of a control group means smaller sample sizes are required and avoids the requirement for large numbers of SAHP treatment to be carried out in the same period. RCT constituted 43.12 % of the study designs and which is considered a reliable design for the evaluation of a medical therapy. However, the quality of these RCTs may have been poor. In our previous systematic review on AHP for stable chronic obstructive pulmonary disease (COPD) and allergic rhinitis (AR) [24, 25], the methodology quality of the included RCTs was generally poor; suggesting that further well-designed RCTs of AHP for COPD and AR is needed. However, we did not conduct an assessment for methodological quality in this present analysis and it is possible that this will be the next phase of our research.

The included studies had 232,138 participants without gender bias and with sufficient representation across different age groups (from 2 to 90 years). 20 % of the studies focused only on children and adolescents. Diseases or conditions treated by SAHP covered 35 categories; the most frequently reported was respiratory diseases, of which asthma was the top one. These findings indicate that SAHP might yield potential advantages for treating respiratory disease with different gender and ages. However, this needs requires a systematic review and meta-analysis to be conducted in order to robustly identify any potential benefits.

It is not difficult to see the diversities in SAHP, such as varying frequency of treatment sessions, length of treatment course and acupoints or herbal ingredients. However, our analysis found that 3 sessions in one *sanfu* period was most commonly used in the included studies. However, it is difficult to confirm whether or not this is the most appropriate treatment arrangement. Indeed, comparisons of different treatment arrangements was the research question for some studies included in our analysis. Hence, we would recommend that future systematic reviews should evaluate frequency of treatment sessions, length of treatment course and other characteristics of SAHP in order to be able to provide stronger recommendations regarding best practice in SAHP.

Outcome evaluation was conducted at the end of SAHP treatment in 373 studies, which we regard unreasonable in order to assess the effectiveness of SAHP. This is because SAHP is used to treat winter conditions and we regard the most appropriate time for outcome assessment should therefore be in the winter or early spring. Furthermore 69.19 % studies applied composite outcome measures rather than internationally-validated outcomes and which limits the interpretations of our findings. Furthermore measures such as quality of life, patient satisfactory and economic outcome were rarely used. Further research should therefore incorporate these in order to evaluate its cost-effectiveness for less affluent societies or for large scale application by commissioning groups.

This bibliometric analysis has several limitations. Firstly severity and course of disease or condition were not extracted in this analysis. Second, did not analyze the journal impact factor and citation per papers. Finally the methodological and reporting quality of included studies was not evaluated.

## Conclusion

To our knowledge, this analysis presents the most complete summary of clinical SAHP studies published to date, and can offer patients, physicians and researchers a more complete assessment of how SAHP is commonly used. Our findings show PICOS (participant, intervention, comparison, outcome and study design) characteristics of SAHP studies in the past 40 years and which can offer researchers an insight into formulating appropriate research questions for the evaluation of SAHP.

Other bibliometric indices also show the research status in this field. We found that research output on SAHP is gradually increasing and that most research is being conducted in China. This suggests that SAHP remains largely unknown in other countries. In addition, 5 studies published on international English-language journals may begin to attract interest from research groups internationally.

## Additional files

Additional file 1:
**Search strategy of each database.** (DOCX 13 kb) Additional file 2:
**The extracted data were as follows.** (DOC 41 kb)
