# Supplementary material for: Characteristics of clinical studies of summer acupoint herbal patching: a bibliometric analysis
Source: BMC Complement Altern Med. 2015 Oct 22;15:381. doi: 10.1186/s12906-015-0905-z (PMC4618877; doi:10.1186/s12906-015-0905-z)
Supplement: Additional file 1: — Search strategy of each database. (DOCX 13 kb) [file 12906_2015_905_MOESM1_ESM.docx]

The extracted data were as follows:

1. Bibliometric data: publication year, origin, journal of publication
2. Study design type
3. Participants: origin, sample size, gender, age, medical condition
4. Intervention: herb ingredients, acupoints selected, treatment session, treatment duration, frequency, control intervention
5. Outcome: follow up, outcome type
