# Supplementary material for: Characteristics of clinical studies of summer acupoint herbal patching: a bibliometric analysis
Source: BMC Complement Altern Med. 2015 Oct 22;15:381. doi: 10.1186/s12906-015-0905-z (PMC4618877; doi:10.1186/s12906-015-0905-z)
Supplement: Additional file 2: — The extracted data were as follows. (DOC 41 kb) [file 12906_2015_905_MOESM2_ESM.doc]

**S1 Search strategy**

**Search strategy of each database:**

**PubMed:**

#1 [Title/Abstract] (“acupoint application”)

#2 [Title/Abstract] (“acupuncture point application”)

#3 [Title/Abstract] (“acupuncture application”)

#4 [Title/Abstract] (“acupoint sticking”)

#5 [Title/Abstract] (“acupoint herbal patching”)

#6 [Title/Abstract] (“sanfutie”)

#7 [Title/Abstract] (“sanfu moxibustion”)

#8 [Title/Abstract] (“sanfujiu”)

#9 [Title/Abstract] (“tianjiu”)

#10 [Title/Abstract] (“Chinese herbal medicine paste”)

#11 [Title/Abstract] (“Chinese herbal medicine plaster”)

#12 [Title/Abstract] (“winter disease treated in summer”)

#13 [Title/Abstract] (“treatment of winter disease in summer”)

#14 [Title/Abstract] (“summer acupoint application treatment”)

#15 #1 OR #2 OR #3 OR #4 OR #5 OR #6 OR #7 OR #8 OR #9 OR #10 OR #11OR #12 OR #13 OR #14

**Cochrane Library:**

[Title/Abstract/Keywords] (“acupoint herbal patching” OR “acupoint sticking” OR “acupuncture application” OR “acupoint application” OR “sanfutie” OR “sanfujiu” OR “Chinese herbal medicine paste” OR “Chinese herbal medicine plaster” OR “treatment of winter disease in summer” OR “winter disease treated in summer”)

**China Network Knowledge Infrastructure (CNKI):**

#1 [Abstract] (“*xue wei tie fu*” (acupoint herbal patching) OR “*xue wei fu tie*” (acupoint herbal patching) OR“*sanfutie*” (common name of acupoint herbal patching) OR “*tian jiu*” (common name of acupoint herbal patching))

#2 [Abstract] (“*dong bing xia zhi*” (winter disease treated in summer) OR “*zhi wei bing*” (preventing disease from exacerbating) OR “*san fu*” OR “*geng ri*” OR “*fu tian*”))

#3 [Abstract] (“*shu*” (rat))

#4 #1 and #2 not #3

**Chinese Scientific Journals Database (VIP):**

#1 [Title/Keywords] (“*xue wei tie fu*” (acupoint herbal patching) OR “*xue wei fu tie*” (acupoint herbal patching) OR“*san fu tie*” (common name of acupoint herbal patching) OR “*tian jiu*” (common name of acupoint herbal patching))

#2 [Title/Keywords] (“*dong bing xia zhi*” (winter disease treated in summer) OR “*zhi wei bing*” (preventing disease from exacerbating) OR “*san fu*” OR “*fu jiu*” OR “*geng ri*” OR “*fu tian*”))

#3 #1 and #2

**Wan Fang database:**

#1 [MeSH terms] (“*xue wei tie fu*” (acupoint herbal patching) OR “*xue wei fu tie*” (acupoint herbal patching) OR “*san fu tie*” OR “*tian jiu*”

#2 [Title/Keywords] (“*sanfutie*” (common name of acupoint herbal patching) OR “*dong bing xia zhi*” (winter disease treated in summer) OR “*zhi wei bing*” (preventing disease from exacerbating) OR “*san fu*” OR “*fu jiu*”))

#3 #1 and #2

**Sino-Med Database:**

#1 [Abstract] (“*xue wei tie fu*” (acupoint herbal patching) OR “*xue wei fu tie*” (acupoint herbal patching) OR“*san fu tie*” (common name of acupoint herbal patching)

#2 [Abstract] (“*dong bing xia zhi*” (winter disease treated in summer) OR “*zhi wei bing*” (preventing disease from exacerbating) OR “*san fu tie*”) OR “*fu jiu*” )

#3 #1 and #2
